# A multi-method evaluation of emotional processing in prospectively predicting suicidal ideation trajectories in adolescents post-psychiatric hospitalization

**DOI:** 10.1017/S0033291725101426

**Published:** 2025-09-12

**Authors:** Lauren A. Haliczer, Yeonsoo Park, Margarid R. Turnamian, Doga Cetinkaya, Sydney A. DeCaro, Evan M. Kleiman, Taylor A. Burke, Richard T. Liu

**Affiliations:** 1 Department of Psychiatry, Massachusetts General Hospital, Boston, MA, USA; 2 Department of Psychiatry, Harvard Medical School, Boston, MA, USA; 3 Department of Psychology, University of Southern California, Los Angeles, CA, USA; 4 Department of Psychology, Southern Methodist University, University Park, TX, USA; 5 Department of Psychology, Rutgers University, New Brunswick, NJ, USA

**Keywords:** adolescent, emotional processing, personal medicine, suicide, trajectory

## Abstract

**Background:**

The months following psychiatric hospitalization are associated with heightened suicide risk among adolescents. Better characterizing predictors of trajectories of suicidal ideation (SI) post-discharge is critical.

**Method:**

We examined trajectories of SI over 18 months post-discharge and emotional processing variables (recognition, reactivity, and regulation) as predictors using a multi-method approach. Participants were 180 adolescents recruited from a pediatric psychiatric inpatient unit, assessed during hospitalization and 3, 6, 12, and 18-months post-discharge. At each time-point, participants reported on SI; at baseline, they completed measures of emotion dysregulation, reactivity, and a behavioral task measuring facial emotion recognition.

**Results:**

A three-group model best fits the data (Chronic SI, Declining SI, and Subthreshold SI groups). The Chronic SI group, compared to the Declining SI group, had greater difficulty identifying children’s sad facial expressions. The Declining SI group compared to the Subthreshold SI group reported greater overall emotion dysregulation and difficulties engaging in goal-directed behavior. No other emotional processing variable was significantly associated with specific SI trajectories.

**Conclusions:**

The findings suggest that difficulties in properly identifying peer emotions may be predictive of resolution of severe SI post-discharge. Furthermore, the results suggest that emotion regulation may be an important target for discharge planning.

## Introduction

Suicide rates are concerningly high among adolescents (Hedegaard & Warner, 2021). Recent years have witnessed a troubling increase in emergency department (ED) and inpatient hospitalization rates for suicidal thoughts and behaviors among youth (Bommersbach, McKean, Olfson, & Rhee, 2023), with percentages of youth mental health hospitalizations for suicidal ideation (SI)/suicide attempts (SA) more than doubling between 2009 and 2019 (Arakelyan et al., 2023). Furthermore, rates of SA in the months after psychiatric hospital discharge remain exceptionally high (Cheek, Goldston, Erkanli, Massing-Schaffer, & Liu, 2020; Czyz & King, 2015; Czyz, Liu, & King, 2012; Yen et al., 2013).

Researchers and clinicians alike have a relatively poor understanding of which patients will be at higher versus lower risk for suicide following discharge from psychiatric hospitalization. This information is necessary to guide appropriate discharge planning and support suicide prevention. Longitudinal studies following psychiatric discharge tend to statistically assume that all individuals will follow the same trajectory, which we know not to be the case. A few studies have explored specific trajectories of SI post-discharge, identifying subgroups of patients with distinct patterns of SI over time. Identifying SI trajectories post-discharge is particularly important because SI has been readily identified as a risk factor of both SA and suicide deaths (Hubers et al., 2018; Large, Sharma, Cannon, Ryan, & Nielssen, 2011; Ribeiro et al., 2016). Findings among adolescent samples have thus far been relatively consistent (Czyz & King, 2015; Giletta et al., 2015; Goldston et al., 2016; Wolff et al., 2018): a subset of patients will continue to experience chronically high SI, a subset of patients with initially elevated SI will experience declining SI, and a subset of patients will experience consistently low SI. The most important distinction for clinicians to make is the difference between the former two groups (i.e. which patients will get better with the passage of time or lower levels of clinical care [e.g. outpatient therapy], and which patients will continue to struggle, necessitating more intensive services [e.g. residential treatment]). Determining this at baseline, when patients with these different eventual trajectories may present similarly, is crucial for treatment and discharge planning in terms of allocation of limited resources and optimally matching patients based on need.

The current literature on trajectories of SI is limited in several ways. First, although there are several studies examining trajectories of SI over time (Giletta et al., 2015; Nkansah-Amankra, 2013; Xiao & Lindsey, 2022) few have followed adolescents post-discharge from acute psychiatric care (e.g. Czyz & King, 2015; Prinstein et al., 2008; Wolff et al., 2018). Second, several studies in this area limit their assessments to three or four time-points (Czyz & King, 2015; Wolff et al., 2018) and/or their follow-up period to 6 months or less (Bloomfield-Clagett et al., 2022; Layrón Folgado et al., 2022; Wolff et al., 2018), with some exceptions that have either examined more time-points or longer follow-up periods (Giletta et al., 2015; Goldston et al., 2016; Nkansah-Amankra, 2013; Whalen et al., 2022; Xiao & Lindsey, 2022). Examining across more time-points and longer follow-up time periods will enable a more nuanced understanding of how SI changes over time, helping to ensure adequate match of inpatients with post-discharge resources (e.g. chronically suicidal patients may benefit from different treatment resources from those patients who experience more temporally circumscribed elevations in SI that resolve over time).

## Emotional processing and SI

### Longitudinal links between emotional processing difficulties and SI

Emotional processing difficulties – particularly in emotion regulation, reactivity, and recognition – may help distinguish between trajectories of SI among psychiatrically acute adolescents. Emotion regulation, an individual’s ability implicitly and explicitly to modulate their emotions (Thompson, 1994), has been associated with SI. For example, greater difficulties accessing emotion regulation strategies predicted greater SI severity 6 months later in a sample of adolescent inpatients (Poon, López, Marie-Shea, & Liu, 2023). Among high school students, feeling unable to handle negative emotions predicted higher SI 6 months later (Choe, Lengua, McFall, & Wyman, 2023). In fact, recent research using real-time monitoring suggests that SI itself may be a maladaptive emotion regulation strategy (Coppersmith et al., 2023). Specifically, there is a bidirectional positive relationship between SI and negative affect in daily life, and use of SI as a form of emotion regulation predicts the frequency and severity of SI at later time-points. Emotion reactivity refers to how readily a person responds to stimuli (i.e. sensitivity), how intensely emotions are experienced (i.e. intensity), and how long those emotions last before returning to baseline (i.e. persistence; Nock, Wedig, Holmberg, & Hooley, 2008). High emotion reactivity is associated with SI over time among adolescents (Wu, Gao, Chen, Zhou, & You, 2023). Similarly, lower positive affect intensity predicts higher suicide risk among adolescent inpatients 6 months later (Yen et al., 2013). Studies have also linked SI to difficulties in emotion recognition, the ability to identify and interpret emotions expressed by another person through various modalities including speech, body language, or facial expressions (Monferrer et al., 2023). For example, youth with SI also demonstrate greater difficulties correctly identifying facial expressions displaying different emotions (i.e. misidentifying angry faces as sad), and the proportion of misclassifications predicts SI onset during a 2-year follow-up (Tsypes, Burkhouse, & Gibb, 2016). Thus, difficulties with emotion regulation, reactivity, and recognition may be candidate predictors of which adolescents will go on to experience persistent SI following psychiatric hospital discharge, and which will improve over time.

### Emotional processing difficulties as predictors of trajectories of SI

A recent study examining SI trajectories across three time-points over 6 months in a clinical adolescent sample (*N =* 104) found that individuals with greater non-acceptance of emotions were more likely to belong to a Chronic SI group than a Declining SI group (Wolff et al., 2018). In a non-clinical sample of bereaved and non-bereaved youth (*N* = 114) who were evaluated at four time-points over 7 years, the Consistent High SI group compared to the Consistent Low SI group demonstrated higher cortisol reactivity, an indicator of emotion dysregulation (Lam, Dickerson, Zoccola, & Zaldivar, 2009), in response to a social stressor task (Shalev et al., 2019). Similarly, Layrón Folgado et al. (2022) explored trajectories of SI over 6 months in a community sample of Spanish college students (*N* = 737) and reported that participants with moderate SI, compared to those with low SI, demonstrated higher levels of negative affect and more emotional suppression. Conversely, participants in the low SI group demonstrated higher levels of positive affect and more cognitive reappraisal. Taken together, more severe SI trajectories tend to be characterized by greater emotion dysregulation and reactivity.

There are several limitations of the modest literature focused on emotional processing variables predicting trajectories of SI. Although some research has explored emotion regulation difficulties as predictors of SI trajectories, it is necessary to more comprehensively examine other relevant components of emotional processing, such as emotion recognition and reactivity. Additionally, studies that do explore difficulties with emotional processing as predictors measure these constructs using only self-report methods (Layrón Folgado et al., 2022; Shalev et al., 2019; Wolff et al., 2018). Using a combination of self-report and task-based behavioral measures of different aspects of emotional processing, in addition to expanding the scope of emotional processing variables under investigation, may help us identify which patients will exhibit more severe SI trajectories post-discharge. Given that emotional processing difficulties can be targeted via clinical intervention (e.g. Dialectical Behavior Therapy [DBT]; Linehan, 1993), this has important suicide prevention and treatment implications.

## Current study

The current study addresses the aforementioned limitations of this literature by first examining trajectories of SI, which is operationalized as ‘thinking about, considering, or planning suicide’ (Klonsky, May, & Saffer, 2016), following inpatient hospitalization in a clinical sample of adolescents over an 18-month period. Second, this study is the first to employ a multi-method approach (i.e. using a combination of self-report and behavioral measures) to comprehensively examine three key emotional processing constructs (i.e. emotion regulation, emotion reactivity, and emotion recognition) as predictors of these trajectories. We hypothesized that (1) consistent with prior literature, there would be three distinct trajectories of SI over time (i.e. chronically elevated SI, initially elevated but declining SI, and chronically low SI) and that (2) emotion dysregulation, emotion reactivity, and difficulties with facial emotion recognition would differentiate among trajectories, with the greatest difficulties in each domain predicting the most persistent SI, and the fewest difficulties in each domain predicting the least persistent SI.

## Methods

### Participants

The sample comprised 180 adolescents (*M*_age_ = 14.89 years; *SD* = 1.35; 71.7% assigned female at birth; 41.7% sexual minority) recruited from a pediatric psychiatric inpatient unit. Inclusion criteria were (1) age between 12 and 17 years and (2) English fluency. Exclusion criteria were (1) psychosis or cognitive impairment that would affect validity of assessments and (2) in the custody of child protective services. The sample’s racial and ethnic distribution was as follows: 17.8% identified their ethnicity as Hispanic; 78.9% identified their race as White, 8.9% as Black, 8.9% as multiracial, and 3.3% as Asian. The median family income range was $50,000–$74,000. The most common psychiatric diagnoses in the sample were major depression (65.5%), generalized anxiety disorder (36.7%), social anxiety disorder (34.4%), attention-deficit/hyperactivity disorder (28.9%), post-traumatic stress disorder (17.2%), and panic disorder (15.0%). At baseline, 68.9% of participants endorsed a lifetime history of non-suicidal self-injury and 58.3% endorsed a lifetime history of suicide attempts.

### Procedure

All study procedures were approved by Rhode Island Hospital’s Institutional Review Board. Participants were approached after consulting with the clinicians on the unit. Informed consent was obtained from participants’ legal guardians prior to enrolment and the adolescents provided assent. After providing informed consent, participants completed assessments at baseline (i.e. during hospitalization), 3-, 6-, 12-, and 18-month post-discharge. At baseline, participants completed measures of SI, emotion dysregulation, emotion reactivity, and a behavioral task measuring facial emotion recognition. SI was assessed at each follow-up.

### Measures

#### Suicidal ideation

The Suicidal Ideation Questionnaire – Jr (SIQ-Jr.) is a 15-item measure assessing severity of past-month SI in adolescents (Reynolds, 1987). Items are rated on a 6-point Likert scale, ranging from 5 = ‘almost every day’, to 0 = ‘not in the past month’ (modified to combine the answer choices ‘I had this thought before but not in the past month’ and ‘not in the past month’ from the SIQ-Jr. for the current study to avoid confounding current SI with past history of SI. Higher scores reflect greater SI severity. A none-zero score indicates some level of current SI (i.e. SI within the past month). The SIQ-Jr. demonstrated sufficient internal consistency at each time-point ( 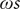 = .94 to .97).

#### Emotion dysregulation

The Difficulties in Emotion Regulation Scale (DERS; Gratz & Roemer, 2004) is a 36-item measure assessing clinically relevant difficulties in emotion regulation. Items are rated on a 5-point Likert scale ranging from 1 (‘almost never, 0–10%’) to 5 (‘almost always, 91–100%’). The DERS consists of six subscales: non-acceptance of emotional responses (i.e. the tendency to have negative secondary emotional responses to one’s negative emotions or non-accepting reactions to one’s distress), difficulties engaging in goal-directed behavior (i.e. difficulties concentrating and accomplishing tasks when experiencing negative emotions), impulse control difficulties (i.e. difficulties remaining in control of one’s behavior when experiencing negative emotions), lack of emotional awareness (i.e. inattention to, and lack of awareness of, emotional responses), limited access to emotion regulation strategies (i.e. tendency to believe that there is little that can be done to regulate emotions effectively once an individual is upset), and lack of emotional clarity (i.e. extent to which individuals know and are clear about the emotions they are experiencing). Higher scores reflect greater dysregulation. Internal consistency for the total score in the current sample was 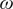 = .93, and for the subscales was as follows: non-acceptance 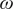 = .89, goal-directed behavior 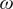 = .89, impulse control 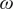 = .89, emotional awareness 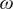 = .80, access to emotion regulation strategies 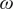 = .87, and emotional clarity 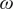 = .81.

#### Emotion reactivity

The Emotion Reactivity Scale (ERS; Nock et al., 2008) is a 21-item measure of emotion reactivity. Items are rated on a 5-point Likert scale from 0 (‘not at all like me’) to 4 (‘completely like me’). The scale provides three subscales: emotion sensitivity (i.e. tendency to respond to a wide range of stimuli), arousal/intensity (i.e. the experience of strong or intense emotions), and persistence (i.e. the experience of emotions for a prolonged period of time before returning to baseline levels of arousal). Higher scores reflect greater reactivity. Internal consistency for the total score in the current sample was 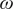 = .96, and for the subscales was as follows: sensitivity 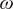 = .90, arousal/intensity 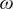 = .91, and persistence 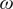 = .83.

#### Emotion recognition

The Diagnostic Analysis of Nonverbal Accuracy (DANVA; Nowicki & Duke, 1994) is a computerized task measuring facial emotion recognition. Participants are shown standardized photos of children and adults displaying happy, sad, angry, or fearful expressions and are instructed to indicate which emotion was expressed. Total error rates are calculated for both the child and adult photos, with happy, sad, angry, and fearful scores calculated for both age groups. Higher scores represent more errors or misattributions. The DANVA has demonstrated good internal consistency, test–retest reliability, and construct validity in prior studies (Nowicki & Duke, 2001).

#### Depression

The Children’s Depression Inventory 2 (CDI 2; Kovacs, 2014) was utilized as a covariate in our analyses given established links between depression and SI (Prinstein et al., 2008), and to ensure any relations found between emotional processing difficulties and SI could not be solely attributed to the presence of depression. The CDI 2 is a 28-item measure; participants are prompted to choose the statement that best describes how they have felt over the past 2 weeks. Items are scored on a scale of 0 (no symptom) to 2 (clinically significant), with higher scores indicating more severe depressive symptoms. Internal consistency for the current sample was 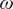 = .92.

### Data analyses

#### Class enumeration

We first generated latent class linear mixture models (also known as growth mixture models) to identify trajectories of SI across the 18-month period using the *lcmm* R package (Proust-Lima, Philipps, & Liquet, 2015). We estimated models with a successive number of classes, ending with a four-class model for the analyses. We determined the optimal model by comparing fit indices across the 1- to 4-class models. Specifically, fit indices included log likelihood (greater is better) and Bayesian information criterion (BIC; lower is better). Additionally, we compared entropy across the four classes, where higher is better, to assess the probability of an individual being assigned to a single class versus belonging to multiple classes. We adjusted for sex assigned at birth, age, and baseline depressive symptoms by adding them as covariates. The SI item was excluded from the depressive symptoms variable to avoid confounding with SI. Given that the aim of the study was to use baseline characteristics to examine different SI trajectories so as to maximize clinical applicability (e.g. to inform clinical decision-making prior to discharge from acute psychiatric care), depression was not considered as a time-varying covariate. More information on covariates can be found in the Supplementary Material.

#### Class differences

We then produced a series of ANOVAs to examine subgroup differences in baseline facial emotion recognition (DANVA), emotion regulation (DERS), and emotion reactivity (ERS). We used the full-scale and subscales for each measure, with one model per scale or subscale. For all significant omnibus differences, we conducted Tukey’s HSD post-hoc analyses, with family-wise error corrections applied.

## Results

### Preliminary results

Descriptive statistics and correlations among study variables can be found in Table 1. At baseline, 164 participants (91%) endorsed some level of SI based on the SIQ-Jr. Retention rates across the time points were 3-month follow-up (95%), 6-month follow-up (88%), 12-month follow-up (88%), and 18-month follow-up (87%). There were no significant group differences between those who were retained at 18-month follow-up compared to those who withdrew or were lost to follow-up on baseline variables (DERS total score, *t*(159) = −0.58, *p* = .564; ERS total score, *t*(159) = −0.01, *p* = .992; CDI 2, *t*(178) = −0.95, *p* = .345), with one exception: individuals retained at 18-month follow-up reported higher average SIQ-Jr. scores at baseline, *t*(178) = −2.18, *p* = .031. There were no differences in attrition based on demographic variables (age, *t*(178) = 0.56, *p* = .577; sex, χ(1) = 0.39, *p* = .533; sexual orientation [sexual minority versus not], χ(1) = 0.15, *p* = .700; race [White versus racial minority], χ(1) = 3.50, *p* = .061).

**Table 1.** Descriptive statistics and correlation matrix of primary study variables

Variable	*M* (*SD*)	Skew	1	2	3	4	5	6	7	8	9
1. BL Emotion regulation (DERS)	111.74 (24.94)	−0.25	–								
2. BL Emotion reactivity (ERS)	49.35 (20.64)	−0.33	.65***	–							
3. BL Emotion recognition – child error rate (DANVA)	.11 (.07)	1.23	.01	−.04	–						
4. BL Emotion recognition – adult error rate (DANVA)	.20 (.80)	0.55	.10	.08	.31***	–					
5. BL Depression (CDI 2)	22.53 (10.96)	−0.06	.53***	.45***	.02	.01	–				
6. BL SI (SIQ-Jr.)	33.14 (21.30)	−0.22	.45***	.44***	−.08	−.08	.64***	–			
7. 3 M SI (SIQ-Jr.)	16.92 (18.54)	0.89	.29***	.29***	−.07	−.02	.41***	.45***	–		
8. 6 M SI (SIQ-Jr.)	12.32 (18.15)	1.37	.30***	.24**	−.10	−.11	.41***	.56***	.59***	–	
9. 12 M SI (SIQ-Jr.)	10.11 (16.35)	1.74	.23**	.20*	.04	.06	.26**	.38***	.37***	.52***	–
10. 18 M SI (SIQ-Jr.)	8.25 (12.84)	1.85	.19*	.21*	.05	−.03	.23**	.27***	.33***	.36***	.59***

Abbreviations: DERS, Difficulties in Emotion Regulation Scale; ERS, Emotion Reactivity Scale; DANVA, Diagnostic Analysis of Nonverbal Accuracy; CDI 2, Children’s Depression Inventory 2; SIQ-Jr. = Suicidal Ideation Questionnaire – Jr; BL, baseline; SI, suicidal ideation; 3 M, 3-month follow-up; 6 M, 6-month follow-up; 12 M, 12-month follow-up; 18 M, 18-month follow-up.

*p < .05;**p < .01; ***p < .001.

### Subgroups of SI trajectories

Table 2 shows the fit statistics and population share by class. A three-class model best fits the data, as it had a higher log likelihood than all other class models, a lower BIC than the one-class model and four-class model, and better entropy than the four-class model. Moreover, the four-class model had a class with only one participant. The overall posterior probability of class assignment, which indicates the probability that individuals were assigned to their most likely class, was .874 (*SD =* .16, range = .41 to .99). Posterior probability for each individual class ranged from .74 to .92.

**Table 2.** Fit statistics and population share by class

Classes in model	Fit statistics			Population share			
	Log lik	BIC	Entropy	Class 1	Class 2	Class 3	Class 4
1	−3470.85	6989.02	1.00	100.00%			
2	−3454.79	6972.68	0.77	15.10%	84.90%		
3	−3447.88	6974.61	0.71	10.42%	66.15%	23.44%	
4	−3447.91	6990.46	0.48	28.65%	60.94%	10.42%	0.55%

*Note*: Population share may not add to 100% in a row due to rounding. Class names across models are arbitrary (e.g. class 2 in the two-class model may not be the same as class 2 in the three-class model). Sex assigned at birth, age, and baseline depressive symptoms were included as covariates.

**Figure 1.**
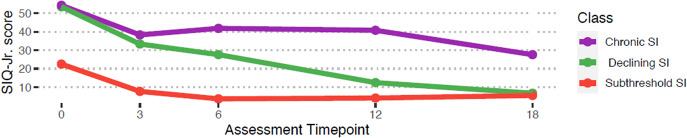
Average trajectories of Suicidal Ideation Questionnaire-Jr (SIQ-Jr.) scores over months of time. *Note.* Chronic SI group: *n* = 19 (10.56%); Declining SI group: *n* = 42 (23.33%); Subthreshold SI group: *n* = 119 (66.11%).

Figure 1 illustrates the trajectories of SI for the three classes across the 18-month period. In terms of class membership, 10.56% (*n* = 19) of the sample fell into a Chronic SI group, which had consistently elevated rates of SI over the 18-month period; 23.33% (*n =* 42) of the sample fell into a Declining SI group, which initially had elevated rates of SI that declined over time; and 66.11% (*n =* 119) of the sample fell into a Subthreshold SI group, which had no or relatively low rates of SI across all time points.

Average scores on the SIQ-Jr. per class at each time point are presented in Table 3. ANOVA analyses using Tukey’s HSD post-hoc test revealed that at baseline, both the Chronic SI and Declining SI groups reported significantly greater average SI scores than the Subthreshold SI group (Omnibus *F* = 6.74, *p* = .001, post-hoc *p*s < .001). Mean differences in SI between the Chronic SI and the Declining SI groups at baseline were not significantly different (*p* = .998). At 18-month follow-up, the Chronic SI group reported significantly greater average SI scores than the Declining SI and Subthreshold SI groups (Omnibus *F* = 34.48, *p* = .001, post-hoc *p*s < .001). Mean differences in SI between the Declining SI and the Subthreshold SI groups at 18-month follow-up were not significantly different (*p* = .840).

**Table 3.** Suicide ideation severity by class across time points

Class	Baseline		3 months		6 months		12 months		18 months	
	Mean	SD	Mean	SD	Mean	SD	Mean	SD	Mean	SD
Chronic SI	53.47	10.95	38.28	17.71	41.83	15.84	40.78	17.59	27.59	14.56
Declining SI	53.24	11.25	33.33	17.12	27.60	19.14	12.44	13.96	6.71	9.42
Subthreshold SI	22.80	17.55	7.69	10.77	3.67	5.76	4.10	9.35	5.49	10.64

Abbreviation: SI, suicidal ideation.

### Subgroup differences in emotion processing variables

Table 4 shows the means, omnibus ANOVA tests, and post-hoc comparisons of baseline variables for all three classes. Levene’s tests to assess homogeneity of variance have been provided in Supplemental Table 1. ANOVA analyses with post-hoc comparisons revealed that the Chronic SI group compared to the Declining SI group had greater difficulty identifying children’s sad facial expressions (*p* = .008). The Declining SI group compared to the Subthreshold SI group reported greater overall emotion dysregulation (*p* = .017) and difficulties engaging in goal-directed behavior when distressed (*p* = .032).

**Table 4.** ANOVA results for baseline emotional processing variables predicting SI trajectories

Variable	Mean (*SD*) by class			ANOVA results			
	Chronic SI (10.56%, *n* = 19)	Declining SI (23.33%, *n* = 42)	Subthreshold SI (66.11%, *n* = 119)	f	p	Post-hoc comparison	η^2^ [95% CI]
Child all error rate	10.09 (6.99)	9.23 (6.80)	11.87(6.89)	1.21	.273		
Child happy	6.14 (5.83)	7.80 (5.21)	8.06 (6.12)	0.51	.477		
Child sad	4.39 (3.96)	1.59 (3.53)	3.39 (4.54)	7.25	.008	Chronic > Declining	.04 [<.01, .12]
Child angry	0.88 (2.79)	0.26 (1.20)	0.88 (2.41)	1.75	.188		
Child fearful	2.05 (3.80)	2.65 (4.46)	3.49 (4.47)	<.001	.994		
Adult all error rate	19.52 (8.34)	18.15 (7.80)	20.43 (8.71)	0.96	.328		
Adult happy	9.65 (8.25)	7.67 (6.83)	9.59 (8.51)	1.33	.250		
Adult sad	5.56 (4.9)	4.37 (4.84)	5.11 (7.58)	0.51	.475		
Adult angry	7.31 (5.87)	7.94 (6.62)	8.06 (7.24)	0.05	.827		
Adult fearful	3.51 (4.23)	4.23 (4.88)	4.47 (5.03)	0.10	.749		
DERS	120.59 (23.06)	127.32 (20.51)	106.99 (23.13)	5.80	.017	Declining > Subthreshold	.03 [<.01, .11]
Non-acceptance	19.21 (7.28)	20.25 (6.81)	15.57 (6.07)	3.09	.081		
Goal-directed behavior	19.39 (4.3)	21.10 (4.13)	17.98 (5.27)	4.68	.032	Declining > Subthreshold	.03 [0.01, .10]
Impulse control	19.83 (6.91)	19.98 (6.02)	17.47 (6.04)	0.76	.386		
Emotional awareness	19.89 (5.31)	20.46 (4.31)	19.21 (5.31)	0.57	.451		
Emotion regulation strategies	30.22 (6.00)	30.75 (6.33)	24.9 (7.22)	3.15	.078		
Emotional clarity	16.33 (5.06)	16.87 (4.03)	14.59 (4.57)	1.61	.206		
ERS	57.71 (19.18)	60.19 (16.11)	45.42 (20.50)	2.69	.103		
Intensity	19.83 (7.66)	21.13 (5.79)	16.13 (7.48)	3.15	.078		
Persistence	27.11 (9.39)	27.64 (8.43)	20.76 (10.04)	1.51	.221		
Sensitivity	10.83 (3.85)	11.32 (3.16)	8.07 (4.16)	3.57	.061		

Abbreviations: DERS, Difficulties in Emotion Regulation Scale; ERS, Emotion Reactivity Scale; SI, suicidal ideation.

## Discussion

Little is known about which psychiatrically acute adolescents will continue to experience persistent SI in the months following discharge from psychiatric hospitalization. This is concerning given the alarmingly high rates of SA among adolescents in this time period (Cheek et al., 2020; Czyz & King, 2015; Czyz et al., 2012; Mirkovic et al., 2020; Yen et al., 2013) and the close association between SI and SA (Ribeiro et al., 2016). Therefore, the current study examined trajectories of SI over an 18-month period following psychiatric inpatient hospitalization among a clinical sample of adolescents; we also examined emotional processing difficulties, using both self-report and task-based measures, as predictors of these trajectories. We anticipated that there would be three distinct SI trajectories and that greater difficulties with emotion regulation, reactivity, and recognition would predict trajectories characterized by chronically elevated SI over time. We found support, albeit mixed, for both of our hypotheses.

Regarding our first aim, a three-class model best fits the data over the 18-month study period. Over 10% of the sample was categorized as Chronic SI, demonstrating consistently high SI rates over time. Just under one-fourth of the sample was categorized as Declining SI, demonstrating initially elevated levels of SI, comparable to those for the Chronic SI group, but that declined over time. Of note, average SI scores in this group declined over time until no longer significantly different from average SI scores in the Subthreshold SI group. Finally, the majority (two-thirds) of participants were categorized as Subthreshold SI, demonstrating relatively low and stable rates of SI over time. This group reported significantly lower average SI scores than both the Chronic and Declining SI groups at baseline. These findings are consistent with those of other trajectory studies with clinical adolescent samples (Czyz & King, 2015; Wolff et al., 2018). Our findings have several important implications. First, as we anticipated, among those who discharge with elevated SI, some patients’ SI will largely resolve over time, while others will continue to experience persistent SI. This highlights the need to identify key variables at baseline (during hospitalization), besides SI, that help distinguish between these groups of patients, and particularly between the two that report clinically elevated SI at discharge. Second, these data suggest that most psychiatrically hospitalized youth will *not* experience clinically significant SI following discharge, which is reassuring.

Regarding our second aim, we found that emotion recognition difficulties differed between these two clinical groups of interest, though findings should be interpreted in relation to the effect sizes and their confidence intervals. Specifically, the Chronic SI group compared to the Declining SI group demonstrated greater difficulties in identifying children’s sad facial expressions. This finding suggests that difficulties recognizing and processing sad faces may be particularly relevant for predicting which adolescents with severe SI at discharge will improve over time versus demonstrate persistently severe SI. Results are consistent with prior, albeit limited, research highlighting facial emotion recognition difficulties among suicidal youth; specifically, youth with a history of SI versus those without are more likely to misclassify full-intensity angry emotions as sad (Tsypes et al., 2016). Of note, there is a dearth of research focused on suicidal adolescent samples and difficulties with recognition of sad faces in particular. Drawing from the depression literature, greater symptom severity was associated with difficulties with both explicit matching of and implicit processing of sad facial expressions (requiring the participant to ignore the target face’s sad emotion and instead recognize the face’s identity; e.g. Porter-Vignola, Booij, Bossé-Chartier, Garel, & Herba, 2021). This suggests that teens with more severe depression may have trouble both recognizing and disengaging from sad faces in particular. However, it should also be noted that the difference between the Chronic SI and Subthreshold group was not significant. Thus, the result may indicate that superior processing of sad faces is associated with favorable outcomes, specifically, decreases in SI over time.

Difficulties processing sad facial expressions may contribute to the exacerbation of suicide risk among clinically acute youth. An important interpersonal function of sadness is to evoke sympathy and helping responses from others (Bonanno, Goorin, & Coifman, 2008). Consequently, difficulties recognizing sadness, and differentiating sadness from other emotions, may be associated with impaired social functioning within this population. For instance, if an adolescent is unable to decipher cues of sadness being conveyed by a close friend, this may lead to a lower likelihood of providing support, which in turn may be interpreted as a lack of caring or understanding. Indeed, social factors, such as interpersonal conflict and isolation, play an important role in suicide risk among adolescents (King & Merchant, 2008). In general, trouble differentiating one’s own negative emotions among adolescents is related to greater negative emotional intensity and frequency and lower beliefs that emotions are malleable. Such difficulties differentiating one’s own emotions may then translate to difficulties identifying specific emotions in others. Taken together, trouble recognizing sad facial expressions predicts persisting, rather than improving, SI in the months following psychiatric hospital discharge among adolescents at elevated risk for suicide; this may be due to a number of downstream inter- and intrapersonal outcomes, such as social conflict and greater negative emotionality, though determining these mechanisms is an important avenue for future research.

Our findings further revealed that the Declining SI group, compared to the Subthreshold SI group, reported greater overall emotion regulation difficulties and greater difficulties engaging in goal-directed behavior when distressed. Our results align with the widely established link between difficulties with emotion regulation and SI broadly and specifically, among adolescent inpatients (Hatkevich, Penner, & Sharp, 2019). These findings suggest the possibility that teaching emotion regulation skills to psychiatrically acute adolescents may accelerate reduction of SI during acute periods of stress (during hospitalization) and during recovery. Emotion regulation skills focused on reducing the likelihood of engaging in impulsive behavior when experiencing negative emotions may be particularly helpful in psychiatrically hospitalized youth (Gratz & Roemer, 2004). For instance, the DBT skill of *opposite to emotion action* involves doing the opposite of a potentially harmful action urge associated with a painful emotion (Linehan, 1993). In the case of a teen who is suicidal and depressed, with a strong urge to isolate from friends and avoid their extracurricular activities, using this skill would involve gently pushing oneself to call a friend or engage in at least one pleasant activity this week. Over time, this should theoretically reduce the intensity of the depressed mood. Indeed, there is preliminary evidence that school-based DBT skills training for early adolescents, which includes the emotion regulation module, is associated with decreases in risky, impulsive behaviors (Zapolski & Smith, 2017).

In addition to these significant findings, it is important to recognize the null findings. First, no significant differences emerged in recognition of adult facial emotions across SI trajectory groups. It is important to note, however, that accuracy in recognizing child facial emotions may be developmentally more consequential and salient for adolescents, given the centrality of peer relationships and the heightened sensitivity to peer evaluation in this developmental stage (Blakemore & Mills, 2014; Somerville, 2013). Our finding that adult emotion recognition was similar across participants, regardless of SI severity, is consistent with a recent EEG study that found adolescents allocated more neural resources in attending to subtle differences in peer-age emotional faces, but not in adult emotional faces (Sandre, Morningstar, Farrell-Reeves, Dirks, & Weinberg, 2022).

Although emotion regulation as a general construct, and specifically the ability to engage in goal-directed behaviors in the face of negative affect, differentiated between the Declining SI and the Subthreshold SI groups, neither other forms of emotion regulation nor emotion reactivity were significant. These findings stand in strong contrast with significant associations for emotion regulation and emotion reactivity as general constructs with SI severity at all time points, which yielded small-to-medium to large effects. Collectively, these findings indicate that the SI trajectory groups are not simply indices of SI severity and thus provide clinically unique information, with different relationships with commonly recognized risk factors. The contrasting pattern of findings highlights the greater challenge of identifying predictors of SI trajectories than SI severity.

This study has some limitations. First, although there were no associations between baseline variables and participant attrition, apart from greater retention of participants with high baseline SI severity, one cannot be absolutely certain that attrition had no effect on the current findings. Future studies could incorporate imputation of missing data to address missingness in data, based on whether data are missing at random or not at random (Enders, 2022). Second, our study used a ‘classify-analyze approach’ (Nylund-Gibson, Grimm, & Masyn, 2019). Despite acceptable average posterior probabilities for individual class assignments (overall = .874; ranging from .74 to .92 for individual classes), the entropy value of .71 for the three-class model falls below the common conventional threshold of .80 (Nylund-Gibson et al., 2019). This suggests that while individuals were confidently assigned to their most probable group, the boundaries between the identified trajectories are not perfectly discrete, potentially introducing inferential ambiguities in subsequent comparisons. This may lead to reduced power to detect significant differences between groups. Although this makes the significant differences between groups all the more notable, future research would benefit from enhanced class differentiation. Third, despite having a longer follow-up period than other studies exploring SI trajectories (Bloomfield-Clagett et al., 2022; Czyz & King, 2015; Layrón Folgado et al., 2022; Wolff et al., 2018; Wu et al., 2023), the present study was limited by a follow-up period of 18 months; future research that extends the period of follow-up and utilizes larger samples will help promote a more nuanced understanding of how SI changes over time among youth at high risk for suicide. Relatedly, future studies may benefit from more frequent evaluations of SI variability, given its association with SA (Witte, Fitzpatrick, Joiner, & Schmidt, 2005). Fourth, although the racial and ethnic composition of the sample largely matches the census data for the local population (Poon et al., 2023), the low presence of racial and ethnic minority participants limits the generalizability of the study findings. For example, there have been studies suggesting that Black youth may have different SI trajectories (Musci et al., 2016). Thus, it is imperative that future studies with similar designs sample from more racially and ethnically diverse populations to determine whether a similar pattern of findings emerges. Fifth, although the inclusion of a task-based behavioral measure of emotion recognition difficulties (i.e. the DANVA) is a unique strength of the current study, future work on SI trajectories may benefit from assessment of a broader range of emotions. Certainly, difficulties recognizing other emotions may be relevant to SI (e.g. shame) (Bentley et al., 2021). Future studies may also leverage technological advances in the assessment of emotion-related constructs (e.g. ecological momentary assessment) (Bettis, Burke, Nesi, & Liu, 2022) to determine how experiences of relevant emotional processes across the hospital stay may be predictive of SI trajectories after discharge. Additionally, as is to be expected in studies of latent classes, the identified groups were not uniform in size. In the current study, the Chronic SI group was smallest, and consequently, there was less statistical power to detect differences for this group than for the Declining SI and Subthreshold SI groups. However, this concern is mitigated to a degree by the fact that the primary significant finding was for the Chronic SI group (relative to the Declining SI group), and when considering that the driving focus of this study was to identify baseline differences that may inform clinical decision-making regarding treatment and discharge planning from acute care settings, for which larger effects are more clinically meaningful and actionable.

The findings from this study have clinical implications. Difficulties identifying children’s sad facial expressions may signal high risk for the experience of persistent SI following psychiatric hospital discharge. Clinical interventions that target not only emotion regulation skills broadly but also emotion identification in others specifically may have promise in suicide risk mitigation. Mindfulness skills, such as those taught in DBT (Linehan, 1993), may be instrumental in helping adolescents better understand specific emotions in terms of their unique presentations and functions, and ultimately, in preventing future suicide in this population.

## Supporting information

Haliczer et al. supplementary material Haliczer et al. supplementary material

## Supplementary material

The supplementary material for this article can be found at http://doi.org/10.1017/S0033291725101426.
