# Supplementary material for: A multi-method evaluation of emotional processing in prospectively predicting suicidal ideation trajectories in adolescents post-psychiatric hospitalization
Source: Psychol Med. 2025 Sep 12;55:e269. doi: 10.1017/S0033291725101426 (PMC13054902; doi:10.1017/S0033291725101426)
Supplement: Haliczer et al. supplementary material [file S0033291725101426sup001.docx]

**Consideration for covariates**

Sexual orientation was not included as a covariate as the subsamples would not be sufficiently large to permit analysis of trajectories. As for non-suicidal self-injury (NSSI) and suicide attempts, theoretical models are consistent in conceptualizing SI as a risk factor for NSSI (e.g., anti-suicide function of NSSI; Klonsky, 2007) and suicide attempts, rather than the other way around. Our analytical plan is consistent with this. Additionally, were we to have had a much larger sample, evaluating whether different SI trajectories are prospectively associated with SA would have been of particular interest.

*Supplemental Table 1. Levene’s Tests for Heteroscedasticity*

| Variable | Levene’s F | *p* |
| --- | --- | --- |
| Child all error rate | 0.86 | .425 |
| Child happy | 0.15 | .864 |
| Child sad | 0.82 | .442 |
| Child angry | 0.38 | .682 |
| Child fearful | 2.06 | .131 |
| Adult all error rate | 1.23 | .296 |
| Adult happy | 0.58 | .559 |
| Adult sad | 2.96 | .054 |
| Adult angry | 2.27 | .107 |
| Adult fearful | 3.36 | .037 |
| DERS | 2.75 | .067 |
| Non-acceptance | 0.32 | .724 |
| Goal-directed behavior | 3.82 | .024 |
| Impulse control | 4.81 | .009 |
| Emotional awareness | 3.09 | .048 |
| Emotion regulation strategies | 0.77 | .465 |
| Emotional clarity | 0.32 | .724 |
| ERS | 0.36 | .701 |
| Intensity | 0.86 | .426 |
| Persistence | 0.39 | .679 |
| Sensitivity | 0.81 | .446 |

*Note.* DERS = Difficulties in Emotion Regulation Scale. ERS = Emotion Reactivity Scale.

The only significant Levene’s test that also had a significant ANOVA was the one for DERS Goal-directed behavior. When reanalyzed using the Welch's test, which does not assume equal variances, the results remained essentially the same (*F* = 5.89, *p* = .024).
